# Sugarcane Vinasse and Glycerol as Potential Carbon Sources for Sulfate-Rich Wastewater Treatment: Mitigating the Impacts of Acid Mine Drainage

**DOI:** 10.1021/acsomega.5c07834

**Published:** 2025-12-08

**Authors:** Elis W. Nogueira, Leandro A. G. Godoi, Pamela T. Couto, Rodrigo B. Carneiro, Paula Y. Takeda, Mirabelle P. Cunha, Bruna D. B. Zampieri, Gunther Brucha, Marcia H. R. Z. Damianovic

**Affiliations:** † Biological Processes Laboratory, São Carlos School of Engineering, 28133 University of São Paulo, Avenida João Dagnone 1100, São Carlos 13563-120, São Paulo, Brazil; ‡ Anaerobic Biotechnology Laboratory, Science and Technology Institute, 74347 Federal University of Alfenas (UNIFAL-MG), Rodovia José Aurélio Vilela, 11999 (BR 267, Km 533), Poços de Caldas 37715-400, Minas Gerais, Brazil; § Laboratory of Chromatography, São Carlos Institute of Chemistry (IQSC), University of São Paulo (USP), Avenida Trabalhador São-Carlense 400, São Carlos 13566-590, São Paulo, Brazil

## Abstract

This study investigated the anaerobic co-digestion of sugarcane vinasse (SV) and glycerol (GOH) as potential carbon sources for treating a sulfate-rich and acidic wastewater (pH 4.0–5.4). The sulfidogenic bioreactor operation was divided into six phases, varying carbon sources and COD/SO_4_^2^–^^ (chemical oxygen demand/sulfate) ratios. In Phases I–III, SV was the sole carbon source (COD/SO_4_^2^–^^ = 1.6–1.2). Phase IV employed co-digestion of SV and GOH, while Phases V and VI used GOH alone, with metals added in Phase VI to simulate acid mine drainage. The highest sulfate reduction (99%) was achieved under co-digestion of SV and GOH for a COD/SO_4_^2^–^^ ratio of 1.4, demonstrating that co-substrate addition can markedly enhance sulfidogenesis. Sulfate reduction was coupled with significant bicarbonate alkalinity generation (>900 mg of CaCO_3_ L^–1^) which stabilized the effluent pH near neutrality (5.3–7.4). In contrast, metal toxicity in Phase VI reduced the biomass performance, highlighting the importance of gradual microbial acclimation under inhibitory conditions. *Desulfovibrio* (5.3%) dominated among sulfate-reducing bacteria, while fermentative genera such as *Zymophilus* (24.8%), *Clostridium* (13.0%), and *Bacteroides* (11.5%) played key syntrophic roles in sustaining sulfidogenesis. Acetate accumulation was associated with pH shifts and incomplete microbial oxidation during later operational stages. Overall, the results demonstrate that co-digestion of SV and GOH is a sustainable and efficient strategy for simultaneous sulfate removal, alkalinity generation, and metal immobilization, providing a promising alternative for the treatment of acid mine drainage and sulfate-rich wastewaters.

## Graphical abstract

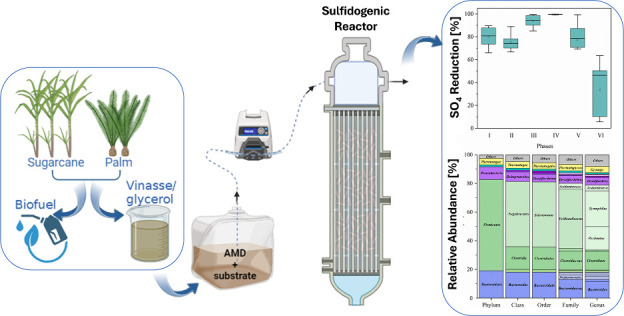

## 1 Introduction

Mining activities have caused severe environmental damage, particularly through the generation of acid mine drainage (AMD), a highly acidic effluent (pH 2–4) rich in dissolved metals (e.g., Fe, Al, Zn, Ni, Cu) and sulfate (up to 3600 mg L^–1^), but poor in nutrients and organic matter.^1,2^ AMD forms when sulfide minerals like pyrite oxidize upon exposure to water and oxygen, producing sulfuric acid and mobilizing metals, whether in mine pits or tailings deposits.^3–5^

Biological treatment appears as an alternative for sulfate reduction and simultaneously alkalinity generation, which are essential for maintaining a pH suitable for microbial activity. Since AMD has very low concentrations of organic matter, the addition of an external electron donor is necessary for its proper treatment. Sulfate in AMD is primarily eliminated by the metabolism of sulfate-reducing bacteria (SRB), which use sulfate as their final electron acceptor. The resulting sulfide from this process can be converted into and recovered as elemental sulfur (S^0^)^6,7^ or can aid in separating dissolved metals in the effluent.^8^ Metals that precipitate as sulfides can be valuable resources if recovered.^9^

Along with different reactor configurations, a variety of electron donors have been employed to treat sulfate-rich wastewater and AMD, including ethanol,^10^ butanol,^11^ lactate,^12^ and other industrial byproducts and wastes. Among the various substrates available, there is increasing interest in using environmental byproducts such as glycerol and vinasse as co-substrates for anaerobic digestion in AMD treatment. Glycerol (GOH), a byproduct used in the pharmaceutical, chemical, food, and cosmetic industries, faces market challenges due to excess production, necessitating new applications to support the sustainability of biodiesel production.^13,14^ GOH serves as a viable alternative carbon source for biological treatment due to its high organic content (1920–5820 g L^–1^), low cost, and nonhazardous classification, which simplifies its management and transportation.^15,16^ Sugarcane vinasse (SV), a byproduct from ethanol production, generates around 11–14 L per liter of ethanol produced.^17,18^ The chemical oxygen demand (COD) of vinasse can range from 19 to 299 g L^–1^.^19–21^ In this way, vinasse, which up to now has no added market value and is a wastewater high in organic matter, nutrients, sulfate, phenolic compounds, and melanoidins,^22^ represents a viable alternative electron donor compared to those commonly discussed in the literature.^11,23,24^ It also offers potential for co-treatment with AMD and other nutrient and carbon-poor wastewater.

Previous studies have shown that SRB-based processes using glycerol as an electron donor achieved high sulfate reduction rates and effective removal of metals such as Zn, Ni, and Cu.^25^ Glycerol, particularly crude glycerol from biodiesel production, effectively reduces sulfate and removes metals As, Cd, Pb, Sb, and Zn from AMD.^26^ It performs well even at low-pH levels, making it suitable for acidic environments.^27^ The addition of glycerol stimulates natural microbial populations, enhancing AMD attenuation through biological sulfate and metals reduction.^28^

The co-digestion of SV and GOH has been investigated to enhance biogas production and improve anaerobic digestion performance.^17,18,29^ In thermophilic anaerobic reactors, this co-digestion has been shown to improve process stability and methane yields even under elevated sulfate concentrations.^17^ Furthermore, the use of glycerol as an alternative to vinasse during the sugarcane off-season has also been investigated to ensure continuous reactor operation.^21^ Although previous studies have tested glycerol or vinasse separately or in multistage systems, the explicit evaluation of co-digestion of sugarcane vinasse (SV) and glycerol (GOH) within a single sulfidogenic reactor—with the objective of simultaneously (i) maximizing biological sulfate reduction, (ii) promoting the precipitation/recovery of dissolved metals as metal sulfides, and (iii) generating alkalinity for in situ pH buffering—remains unexplored. This single-reactor, integrated approach is the core innovation of the present work: it tests whether two abundant biofuel byproducts can be used to meet multiple treatment goals (sulfate reduction, metal removal, and alkalinization) in a compact, operationally simpler system compared to multistage alternatives.

## 2 Methods

### 2.1 Bioreactor Setup

A sulfidogenic DFSBR with a useful volume of 2.0 L was operated under mesophilic conditions (30 °C), maintained in a temperature-controlled chamber for 236 days to carry out the co-digestion of AMD, SV, and GOH. The support material was composed of low-density polyethylene cylinders, arranged on four rods within the central column as described by Nogueira et al.^9^ Fixed-structured-bed reactors offer several advantages due to their ability to achieve a high cell retention time (CRT) and maintain elevated concentrations of active biomass. They also result in low suspended solids content in the effluent, high performance, and operational stability, while avoiding clogging issues that are common in packed bed reactors.^30,31^ The choice of support material for use in the DFSBR is justified by the benefits of biofilm development on carrier media, which enable high CRT and elevated biomass concentrations. These high CRTs favor the enrichment of slow-growing microorganisms and can enhance resistance to toxic and recalcitrant compounds.^32^

The inoculation process followed the protocol proposed by Godoi et al.^33^ utilizing the sludge recirculation technique in the sedimentation zone for 7 days. The inoculum used was sourced from a pilot-scale mesophilic UASB reactor adapted for the treatment of SV. The SV used in this study was collected from the Rio Pardo Distillery, located in Cerqueira César, São Paulo, Brazil. This industrial vinasse was concentrated by evaporation, resulting in a concentration of approximately 120 g of COD L^–1^. The SV characterization is presented in the Supporting Information (Table S2), and the analytical procedures are detailed in Section 2.3. Pure glycerol (99.5% purity) with an average density of 1.26 g cm^–3^ was used as the carbon source (Phases IV–VI). Owing to its high COD content (approximately 1.5 g L^–1^), the glycerol was diluted to the target COD concentrations before being fed into the reactors. In contrast to crude glycerol, which often contains impurities such as inorganic salts, residual fatty acids, and glycerides that may inhibit microbial activity, pure glycerol provides a clean and readily available carbon source, enhancing anaerobic performance.^18,34^

### 2.2 Operational Phases

The operation of the DFSBR was divided into six distinct phases, as summarized in Table 1. To promote favorable conditions for SRB, the COD/SO_4_^2^–^^ ratio was maintained above the stoichiometric value of 0.67, which represents the theoretical amount of COD required to reduce 1 g of SO_4_^2^–^^ to H_2_S.^35^ This ratio is considered optimal for SRB growth and efficient sulfate reduction. However, previous studies have shown that higher ratios can enhance system performance.^36–38^ For instance, Mahesh et al.^39^ and Cunha et al.^36^ reported that COD/SO_4_^2^–^^ ratios between 1.0 and 1.2 yielded the highest COD and sulfate removal efficiencies. Phase I aimed to replicate the operational conditions described by Godoi et al.,^33^ applying an organic loading rate (OLR) of 4.8 g of COD L^–1^ day^–1^ and a sulfate loading rate (SLR) of 3.0 g of SO_4_^2^–^^ L^–1^ day^–1^. In Phase II, the focus was on enhancing sulfidogenesis by reducing the extent of the OLR through dilution of SV, thereby decreasing the COD/SO_4_^2^–^^ ratio from 1.6 ± 0.3 to 1.2 ± 0.2. Phase III maintained this COD/SO_4_^2^–^^ ratio but further reduced both the OLR and SLR to evaluate reactor performance under lower loading conditions. Phase IV marked the transition from SV to GOH as the carbon source, starting with a mixture of 20% GOH and 80% SV (v/v) and then progressing to a 50:50 (v/v) ratio. This transition was completed in Phase V, where pure GOH became the sole electron donor. Until Phase IV, SV was the only sulfate source in the synthetic wastewater. From Phase V onward, Na_2_SO_4_ was supplemented to maintain a COD/SO_4_^2^–^^ ratio of 1.4 ± 0.1. In Phase VI, a metal solution was introduced to simulate AMD, using FeSO_4_·7H_2_O, ZnCl_2_, CoCl_2_·2H_2_O, NiCl_2_·6H_2_O, and CuSO_4_·5H_2_O, following the composition proposed by Nogueira et al.^40^ The respective influent concentrations of Cu, Fe, Co, Ni, and Zn were 2.15 ± 0.07, 52.98 ± 1.73, 3.30 ± 0.03, 4.18 ± 0.17, and 8.17 ± 0.32 mg L^–1^. The synthetic wastewater was prepared using raw SV, and its characterization is detailed in Table S2, with COD concentrations adjusted to match the values specified for each phase in Table 1. Given the dynamic and nonlinear behavior of plug-flow reactors, continuous pH regulation presents significant operational challenges. Therefore, the reactor was operated without active pH regulation. The influent pH was primarily determined by the addition of SV; when GOH was used as the sole carbon source, the pH was adjusted by using HCl.

**Table 1:** Experimental Conditions Applied in the DFSBR

	for given phase					
param	I	II	III	IV	V	VI
operation time (days)	1–50	51–90	91–114	115–140	141–183	184–236
carbon source^a^	SV	SV	SV	SV + GOH	GOH	GOH
OLR (g L^–1^ day^–1^)	4.8 ± 0.7	3.5 ± 0.2	1.6 ± 0.6	1.8 ± 0.4	2.5 ± 0.4	1.8 ± 0.4
SLR (g L^–1^ day^–1^)	3.0 ± 0.4	3.0 ± 0.4	1.4 ± 0.6	1.4 ± 0.6	1.8 ± 0.2	1.6 ± 0.4
COD soluble (g L^–1^)	4.3 ± 0.6	3.1 ± 0.2	1.3 ± 0.1	1.5 ± 0.2	2.2 ± 0.2	1.7 ± 0.1
SO_4_^2^–^^ (g L^–1^)	2.7 ± 0.4	2.7 ± 0.3	1.1 ± 0.3	1.1 ± 0.3	1.6 ± 0.1	1.5 ± 0.2
COD/SO_4_^2^–^^	1.6 ± 0.3	1.2 ± 0.2	1.2 ± 0.3	1.4 ± 0.1	1.4 ± 0.1	1.2 ± 0.1
pH influent	4.7	4.9	5.4	4.9	3.4	4.0

a SV, sugarcane vinasse; GOH, glycerol.

### 2.3 Analytical Methods and Calculations

The analyses of COD, sulfide, sulfate, pH, and total solids were performed following the procedures outlined in ref 41. The variation in electron flow due to COD oxidation by SRB was calculated based on the work of Lens et al.^35^ Total alkalinity, partial alkalinity (PA), intermediate alkalinity (IA), and alkalinity due to VFA were measured using the method described by Godoi et al.^42^ The concentrations of VFAs, specifically acetate, propionate, butyrate, and isobutyrate, were quantified using a Shimadzu GC-2010 gas chromatograph (Shimadzu Scientific Instruments, Columbia, MD, USA) equipped with a flame ionization detector (FID) and an HP-INNOWAX capillary column (30 m × 0.25 mm × 0.25 μm). The analytical procedure followed the validated method described by Adorno et al.,^43^ which is widely applied for monitoring anaerobic processes due to its reliability and sensitivity. For headspace analysis, 2 mL of the sample was transferred to a 10 mL sealed vial containing 1 g of NaCl, 70 μL of isobutanol, 100 μL of crotonic acid, and 200 μL of 2 M H_2_SO_4_. The vials were heated at 100 °C for 13 min prior to injection.^43^

Prior to COD and sulfide analyses, a protocol outlined by Poinapen et al.^44^ was followed to prevent the loss of dissolved sulfide. This protocol involves adding three drops of 10 mol L^–1^ NaOH to the sample to convert H_2_S to HS^–^ and S_2_^–^, ensuring that the sulfide remains in its dissociated form, thereby preventing volatilization losses. For the COD analysis, three distinct fractions of the effluent were measured: total soluble COD, COD due to dissolved sulfide (COD_sulfide_), and COD due to dissolved organic matter (COD_organic matter_). To determine the organic COD, excess zinc sulfate (ZnSO_4_) was added to precipitate the liquid sulfide as zinc sulfide (ZnS). The difference between dissolved COD and organic COD corresponds to the fraction of COD_sulfide_.^44^ Additionally, to determine the speciation forms of sulfide (H_2_S and HS^–^) and to assess the alkalinity due to sulfide and bicarbonate in the DFSBR effluent, the calculations proposed by Godoi et al.^22^ and Parsaee et al.^45^ were applied.

Total metal concentrations in the wastewater samples were measured using inductively coupled plasma optical emission spectroscopy (ICP-OES) with a Thermo Scientific iCAP 6000 Series instrument (Germany). Approximately 30 mL of each sample was collected in high-density polyethylene (HDPE) bottles, acidified with 2% (v/v) HNO_3_, and stored at 4 °C until analysis. Prior to quantification, samples underwent aqua regia digestion in a heating block (TE40-05, Tecnal) at 150 °C for 4–6 h, following standard methods.^41^

Samples from the bottom of the DFSBR were collected on days 20 and 50 (Phase I), 70 (Phase II), 98 (Phase III), 117 (Phase IV), 183 (Phase V), and 190, 201, and 236 (Phase VI) and analyzed for the concentrations of total, suspended, fixed, and volatile solids (TS, SS, FS, and VS, respectively).^41^

To characterize the SV, analyses were performed for COD, BOD_5_ (at 20 °C), various solid fractions, soluble carbohydrates, phenolic compounds, ammoniacal nitrogen, total Kjeldahl nitrogen, nitrite, nitrate, phosphorus, and sulfate, following the standard methods.^41^ Metal concentrations—including cadmium, calcium, copper, lead, iron, magnesium, manganese, nickel, potassium, sodium, and zinc—were determined using atomic absorption spectrophotometry in accordance with ref 41.

### 2.4 Microbial Community Analysis

To assess the structure and resilience of the microbial community, biomass samples were collected from the reactor at the end of Phase VI. This sampling point was chosen to capture the cumulative effects of all operational changes, particularly the influence of metal addition and low pH, representative of an AMD simulation scenario. DNA was extracted and purified using the Wizard Genomic DNA Purification Kit (Promega), followed by PCR amplification of the V4 region of the 16S rRNA gene for both bacteria (primers 515F-806R) and archaea (primers 341F-785R). The PCR products were purified using AMPure XP beads and used for library preparation. Sequencing was performed using the Illumina MiSeq platform at MR DNA (Shallowater, TX, USA), following standard protocols. The raw sequencing data were processed through MR DNA’s proprietary analysis pipeline. This included removal of barcodes and primers, exclusion of sequences shorter than 150 bp, filtering of sequences with ambiguous base calls or homopolymer runs > 6 bases, denoising, chimera removal, and OTU (operational taxonomic unit) clustering at 97% similarity (3% divergence). Taxonomic classification was performed using BLASTn against a curated reference database (GreenGenes, RDPII, and NCBI). Microbial diversity was evaluated using the Shannon (H′), Simpson (1-D), Margalef (DMg), and Chao1 richness indices. All diversity calculations were performed using PAST software (Paleontological Statistics Software, version 4.03). Sequencing data have been deposited in the NCBI Sequence Read Archive under BioProject accession no. PRJNA1234676.

### 2.5 Statistical Analysis

Statistical analyses were conducted using Statistica, version 14, with a significance level set at 5%. Data normality was assessed using the Kolmogorov–Smirnov and Lilliefors test for the overall data set and the Shapiro–Wilk test for individual group analyses. When the normality assumption was satisfied, statistical differences among phases were evaluated using one-way analysis of variance (ANOVA), followed by Tukey’s post hoc test to identify specific differences between groups. When normality was not assumed, nonparametric tests (Mann–Whitney) were applied. Differences were considered statistically significant at *p* ≤ 0.05, corresponding to a 95% confidence level.

## 3 Results and Discussion

### 3.1 DFSBR Performance in COD Removal and Sulfate Reduction

As the COD/SO_4_^2^–^^ ratio decreased from Phase I to Phase II due to a drop in influent COD relative to SV, the average efficiency of organic matter removal decreased from 58.1 ± 10.2 to 51.2 ± 5.6%, as shown in Figure 1 (Table S1). Despite this reduction, the results exhibited less variability and there was no statistically significant difference between Phases I and II (*p*-value > 0.05). However, a statistically significant difference was observed between Phase II and Phase III (*p*-value < 0.05). With further reduction in the OLR and SLR, the average COD removal efficiency increased to 63.5 ± 10.5%, likely due to the higher acetic acid concentration in Phase II (detailed in Section 3.4). This efficiency stabilized at 61.0 ± 15.0% in Phase IV when GOH was introduced into the system. In Phase V, with GOH serving as the sole electron donor, the average removal efficiency further improved to 67.0 ± 12.0%. However, following the addition of metals in Phase VI, there was a notable decline in the COD removal efficiency, dropping to 35.0 ± 18.0% (Table S1).

**Figure 1:**
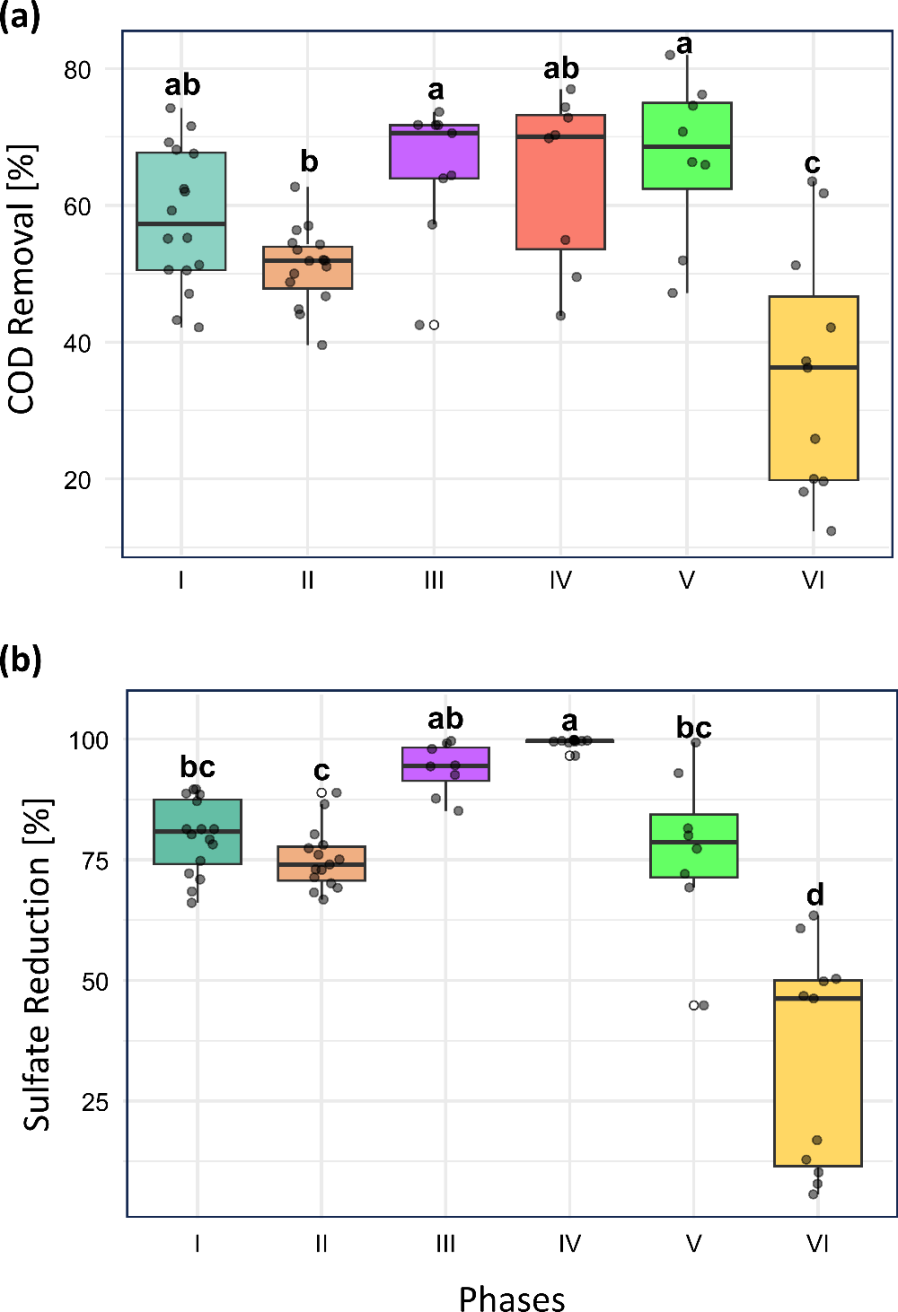
Box plot of the COD removal efficiency (a) and sulfate reduction (b) in each operational phase. Values are listed in Table S1. Different letters above the boxes indicate statistically significant differences among phases (*p* < 0.05) according to one-way ANOVA followed by Tukey HSD test.

Regarding sulfate reduction (Figure 1), the average efficiency slightly decreased from 79.8 ± 7.7% in Phase I to 75.2 ± 6.3% in Phase II. This change, which corresponded with the decrease in organic matter input, was not statistically significant (*p*-value > 0.05). In Phases III and IV, the efficiency stabilized above 90%. The transition from SV to GOH in Phase IV did not negatively affect reactor stability or efficiency. However, during Phase V (100% GOH), a gradual decline in sulfate reduction was observed, decreasing from 99% at the beginning to 69% by the end of the phase. With the addition of dissolved metals in Phase VI, sulfidogenesis was significantly impaired (*p*-value < 0.05), and the average removal efficiency dropped sharply to 33%. From day 215 onward, values fell below 20%. This performance decline in Phase V may be attributed to the rapid and nearly complete conversion of GOH into acetate (Figure 4), due to its higher biodegradability compared to SV. The accumulation of VFA, especially acetate, likely contributed to the observed sulfate reduction drop by decreasing the influent pH and shifting the sulfide speciation in the system (Sections 3.2 and 3.4).

While the simultaneous use of SV and GOH in Phase IV proved to be the most effective combination for sulfate reduction, achieving 99% efficiency, using GOH alone as the carbon source showed instability. Since GOH is a component of SV, using fresh SV as an electron donor offers advantages due to its high organic load and concentration of nutrients such as P, N, and K^22,45^ which are absent in pure GOH.

The introduction of metals without prior biomass adaptation in Phase VI likely had a significant impact on the microbial community, resulting in decreased COD and sulfate removal efficiencies. Metals in solution can inhibit the activity of SRB by acting as barriers that prevent the exchange of solutes across microbial cell membranes.^46,47^ Specifically, the accumulation of approximately 5 mg of Cu L^–1^ and 10 mg of Zn L^–1^ can impair sulfate reduction in AMD treatment.^47,48^ However, Cunha et al.^49^ observed that a gradual increase in metal concentration improved both COD removal and sulfate reduction efficiencies. Similarly, Gallegos-Garcia et al.^50^ reported that the gradual addition of metals such as Fe, Zn, and Cd did not negatively affect sulfate reduction or COD removal. However, it did have a negative impact on effluent pH, which decreased from 6.0 to 5.2, and on bicarbonate alkalinity, which dropped from 1314 ± 19 to 0 mg L^–1^ after the addition of metals.

Villa-Gomez et al.^51^ found that the influent pH of the medium significantly influenced the treatment efficiencies of wastewater containing metals. A drop in influent pH from 7 to 4 negatively impacted the COD and sulfate removal efficiencies, which did not exceed 30%. Papirio et al.^52^ reported that in inverse fluidized-bed reactors, the biological process failed when the feed pH was intentionally decreased from 5.0 to 3.0. Sulfate reduction efficiency declined from 49% in Period IV to 2% in Period V, indicating near-complete inhibition of SRB activity. Concurrently, COD removal efficiency and effluent pH dropped to 6% and 3.0, respectively, demonstrating that lowering the feed pH to 3.0, even at a COD/sulfate ratio of 4.0, resulted in process failure.^52^ Conversely, Vieira et al.,^53^ using batch reactors, observed that sulfate removal efficiency increased from 38.5 to 52.2% as the pH decreased from 7.0 to 4.0, while COD removal reached 99%. The presence of metals (Fe, Zn, and Cu) further enhanced sulfate removal to 82.2%.

Xingyu et al.^54^ operated a low-pH sulfidogenic UASB reactor using real, undiluted AMD and achieved 38% sulfate removal. When the influent was diluted, sulfate removal increased to 60–80%. Similarly, Rodriguez et al.^55^ reported over 75% removal of both COD and sulfate using real AMD in horizontal-flow anaerobic immobilized biomass reactors, operating at pH 3.0–4.0, with optimal performance observed at a COD/SO_4_^2^–^^ ratio of 0.67.

In the study reported by Nogueira et al.,^56^ a structured fixed-bed bioreactor was operated for 385 days with gradually increasing proportions of real AMD. During Phase I, COD removal averaged 60 ± 14% with synthetic AMD but decreased to 50 ± 26% when 20% real AMD was introduced in Phase II. With 50% real AMD (Phase III), COD removal improved to 71 ± 10%, before declining to 65 ± 22% in Phase IV at 75% AMD. Sulfate reduction exhibited a similar trend, reaching 37 ± 20% in Phase II after microbial adaptation and increasing to 44 ± 13% in Phase III. Although efficiencies temporarily peaked at 69% in Phase IV, a sharp decline was observed after sludge discharge, likely due to the loss of active sulfate-reducing biomass.

The use of GOH in the treatment of sulfate-rich wastewater, whether for sulfate reduction or metal removal, has proven to be efficient.^25,57^ However, in these studies, the added copper was removed via selective precipitation, meaning that the effluent containing the metal was treated in a separate compartment, preventing direct contact between the metal and the microbial biomass.

Some studies have correlated the loss of sulfate removal efficiency with the addition of high concentrations of metals without adequate adaptation of the microbial population.^47,58,59^ This situation leads to the accumulation of organic compounds due to incomplete oxidation by SRB and causes changes in the effluent pH. The data from the present study support these findings.

These results highlight the importance of gradual substrate adaptation and load management to prevent process failure. In practical applications, a stepwise acclimation strategy, where the proportion of GOH is progressively increased while maintaining stable COD/SO_4_^2^–^^ ratios and alkalinity, can effectively minimize sudden metabolic shifts and allow microbial communities, particularly sulfate-reducing and methanogenic populations, to adjust their enzymatic and regulatory mechanisms. Alternatively, a two-stage treatment strategy could be implemented, in which bioreactors enriched with mixed communities of acidophilic sulfate-reducing bacteria are used to promote the selective precipitation of transition metals as sulfides.^8,60^ This approach, as demonstrated by Ñancucheo and Johnson,^8^ effectively reduces metal toxicity in sulfate-rich systems by decoupling metal removal from sulfidogenic activity, thereby mitigating the inhibitory effects observed in the present study.

From an engineering perspective, the use of SV + GOH presents both economic and operational benefits when compared with conventional electron donors such as ethanol or lactate. Both SV and GOH are low-cost byproducts of the sugarcane and biodiesel industries, respectively, offering a sustainable and regionally abundant alternative to commercial organic substrates.^18,21^ The complex matrix of SV provides essential nutrients and trace elements that support microbial growth and buffer reactor conditions,^9,33^ while GOH supplies a readily available source of reducing power, promoting high sulfate reduction rates when properly managed.^56^ In contrast, ethanol and lactate are easily metabolized and can ensure stable sulfidogenesis,^49^ but their higher cost and safety requirements limit their large-scale applicability. Therefore, the combination of SV and GOH can be considered a promising strategy for sulfate-rich wastewater treatment, provided that gradual acclimation and pH control are implemented to prevent excessive VFA accumulation and maintain reactor stability under dynamic conditions.

From a practical and regional perspective, and considering Brazil’s current energy scenario, the co-treatment of AMD, SV, and GOH is highly feasible in regions where mining activities overlap with ethanol and biodiesel production. Given the markedly different organic contents of these three substrates—from extremely low in AMD to very high in SV and GOH (as detailed in the Introduction)—their combined treatment is technically justified, as it promotes complementary nutrient and carbon balancing. The transportation of SV and GOH from ethanol and biodiesel plants represents only a minor cost component, especially since similar logistics are already in place for the transport of alkalinizing agents used in conventional physicochemical treatment. These costs can be further minimized through return logistics, utilizing existing delivery routes, and infrastructure.

Beyond the environmental and operational benefits, this biological process also presents a clear economic advantage compared with the conventional physicochemical treatment currently employed for AMD remediation. For example, at the Brazilian Nuclear Industries facility, in 2018, the company spent approximately 1.8 million Brazilian reais (approximately 430 thousand U.S. dollars) on calcium hydroxide to increase the pH of AMD above neutrality prior to discharge, as required by Brazilian environmental regulations.^9^ In addition to the high reagent and sludge management costs, such abiotic remediation systems generate large volumes of chemical sludge, whereas biological sulfidogenic processes generate significantly less solid waste and allow metal recovery via sulfide precipitation.^61^ Therefore, the integration of SV and GOH as carbon sources in anaerobic sulfidogenic reactors represents not only an environmentally sustainable but also a cost-effective and circular solution capable of reducing chemical consumption and sludge generation while simultaneously enabling the recovery of valuable metals from AMD.

### 3.2 Sulfide Production and Metal Removal

Regarding the biosulfide produced, the effluent average concentration of total dissolved sulfide was 462 ± 180 mg L^–1^ in Phase I and 462 ± 112 mg L^–1^ in Phase II. The averages for Phases III, IV, V, and VI were 342 ± 99, 410 ± 83, 313 ± 150, and 72 ± 52 mg L^–1^, respectively (Figure 2). Sulfide predominantly existed in the form of HS^–^, representing 82% of the total in Phase I and 75% in Phase II. In Phases III, IV, and V, HS^–^ accounted for 69, 75, and 63% of the total sulfide, respectively. In Phase VI, only 8.6% of the total sulfide was in the form of HS^–^ due to the drop in effluent pH. In the form of H_2_S, which is the most toxic to microorganisms, concentrations of H_2_S exceeded 110 mg L^–1^ only during Phases II and V, a value considered inhibitory for both archaea and bacteria.^62^ Cunha et al.,^36^ using a UASB reactor, observed a drop in biological treatment efficiency when the total sulfide concentration exceeded 270 mg L^–1^, with approximately 90 mg L^–1^ in the form of H_2_S.

**Figure 2:**
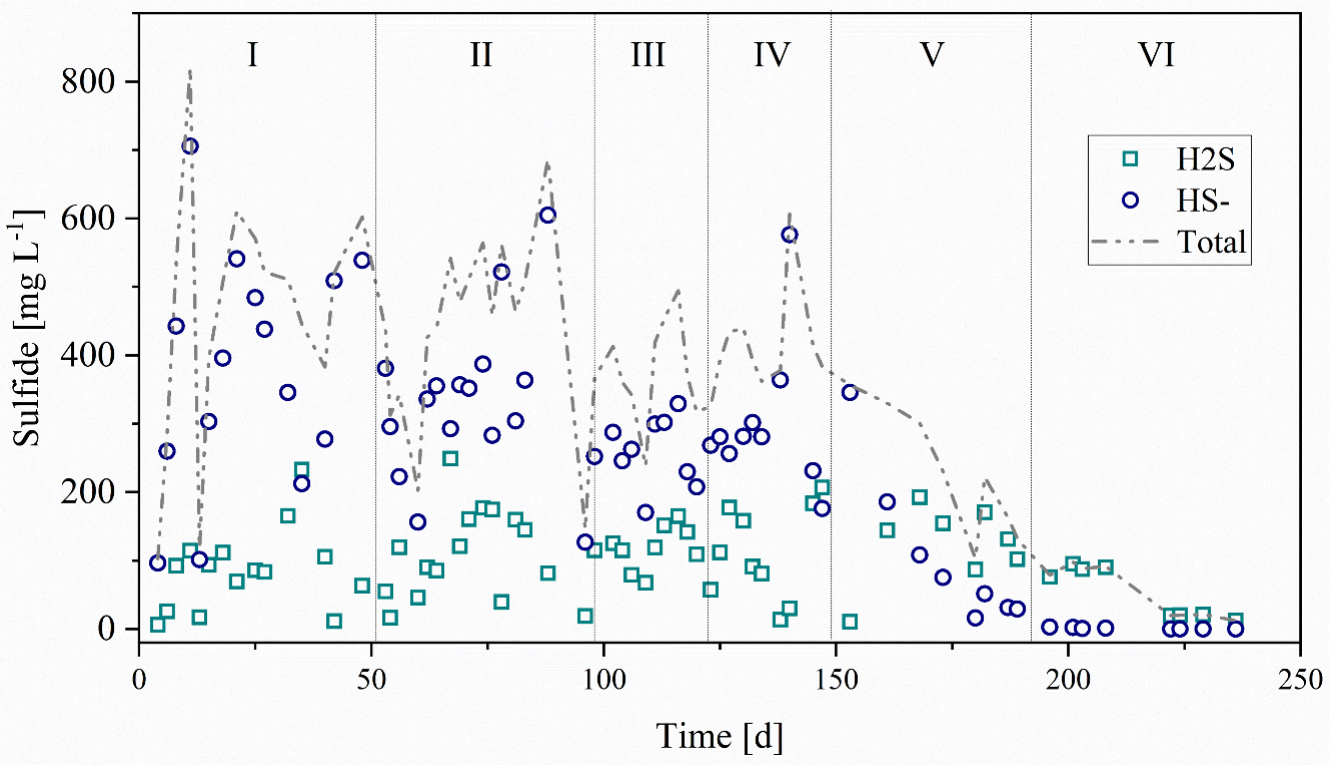
Total sulfide concentration (—), hydrogen sulfide (H_2_S) (□), and bisulfide anion HS^–^ (○) during each operational phase.

Previous studies suggest that free H_2_S concentrations between 50 and 250 mg L^–1^ can inhibit anaerobic digestion.^62^ The critical threshold concentration of H_2_S for SRB is generally reported at levels above 125 mg L^–1^,^63^ while for methanogens, inhibitory concentrations range from 50 to 400 mg L^–1^.^47,64^ Total inhibition of SRB metabolism has been observed at H_2_S concentrations exceeding 500 mg L^–1^ in batch reactors.^65^ For methanogens, the inhibitory concentration of total dissolved sulfide ranges from 100 to 800 mg L^–1^.^64^ In this study, hydrogen sulfide concentrations remained within these inhibitory thresholds throughout the operational period. Therefore, the observed performance decline and signs of toxicity in the sulfidogenic biomass during Phase V can be attributed to the combined effects of acidic conditions, VFA and hydrogen sulfide accumulation.^66^

Regarding metals, the data presented in Table 2 demonstrate the effectiveness of the treatment process in removing various metals (Co, Cu, Fe, Ni, and Zn). The high removal efficiency of Cu may be attributed to the relatively low initial concentration and its strong tendency to precipitate within the treatment system. The gradual increase in removal efficiencies, particularly for Co, Ni, and Zn, indicates that the treatment system would require time to stabilize and optimize its performance. The observed trends at the beginning of the Phase VI could be due to microbial adaptation, where SRB became more effective at generating sulfides for metal precipitation. However, the slight decline in Fe removal on day 196th may point to potential limitations in the system such as accumulation of precipitates or inhibition by metal concentrations in the system. Despite this, the overall results are promising, demonstrating the system’s capability to handle mixed-metal wastewater effectively.

**Table 2:** Removal Efficiencies and Influent/Effluent Metal Concentrations during Phase VI

		day 188		day 189		day 196	
metal	influent (mg L^–1^)	effluent (mg L^–1^)	removal (%)	effluent (mg L^–1^)	removal (%)	effluent (mg L^–1^)	removal (%)
Co	3.30 ± 0.03	2.32 ± 0.35	29.50	0.88 ± 0.00	73.21	0.65 ± 0.00	80.36
Cu	2.15 ± 0.07	0.72 ± 0.11	66.66	0.45 ± 0.01	79.31	0.37 ± 0.00	82.98
Fe	52.98 ± 1.73	16.51 ± 2.49	68.84	1.62 ± 0.02	96.95	6.14 ± 0.08	88.41
Ni	4.18 ± 0.17	2.93 ± 0.44	29.88	1.02 ± 0.01	75.62	0.72 ± 0.00	82.82
Zn	8.17 ± 0.32	6.43 ± 0.91	21.29	2.36 ± 0.01	71.07	2.12 ± 0.25	74.07

The primary mechanism involves SRB producing hydrogen sulfide, which reacts with metal ions to form insoluble metal sulfides that precipitate from solution (Reactions [Disp-formula eqR1] and [Disp-formula eqR2]).^67^ Sun et al.^68^ demonstrated >99.9% removal of ferric, zinc, and copper ions using a novel sulfur reduction system, where zinc and copper were removed via biogenic hydrogen sulfide precipitation (Reaction [Disp-formula eqR2]), while ferric was primarily removed through alkali precipitation (Reaction [Disp-formula eqR3]).^68–70^ Similar mechanisms are expected to occur in the DFSBR, where Cu, Zn, Ni, Fe, and Co can precipitate as metal sulfides (Reaction [Disp-formula eqR2]),^49,69,71^ while Fe may also precipitate as hydroxide species under more alkaline conditions (Reaction [Disp-formula eqR3]).^68,69^ The general reactions involved are as follows:

R1 $${{\mathrm{SO}}_{4}}^{2-}+\mathrm{nutrients}+H_{2}O\to H_{2}S+{{\mathrm{HCO}}_{3}}^{-}$$

R2 $$M^{2+}+H_{2}S\to \mathrm{MS}+2H^{+}$$

R3 $$M^{2+}+2OH^{-}\to M{\left(\mathrm{OH}\right)}_{2}↓$$

Additional mechanisms include biosorption and intracellular metal accumulation.^72^ Low concentrations of metals can stimulate microbial growth and support specific metabolic pathways.^73^ However, when metal concentrations exceed certain levels, they can precipitate as metal sulfides, reducing both the metal and sulfide concentrations, thus decreasing their toxicity to microorganisms.^74^ It is important that this precipitation does not significantly affect the alkalinity, which could disrupt the treatment process due to the formation of metal sulfides.^33^

To mitigate both sulfide toxicity and metal precipitation, two-stage reactor systems appear to be a promising solution.^38^ According to Cunha et al.,^36^ Uçar,^75^ and Yilmaz et al.,^76^ utilizing dissolved sulfides generated during sulfate reduction and converting them into gas for transfer to a secondary reactor represents one of the most effective methods for metal precipitation in such systems. This approach not only improves metal removal but also enhances sulfide management, thereby increasing the overall efficiency of the treatment process.

### 3.3 Alkalinity and pH

Bicarbonate alkalinity average was 1375 ± 274 mg L^–1^ in Phase I and 1275 ± 196 mg L^–1^ in Phase II, showing no significant difference (*p*-value > 0.05). In Phases III and IV, bicarbonate alkalinity remained above 900 mg L^–1^, accounting for more than 90% of the total alkalinity. In Phase V the bicarbonate fraction decreased to 575 ± 270 mg L^–1^ (Figure 3).

**Figure 3:**
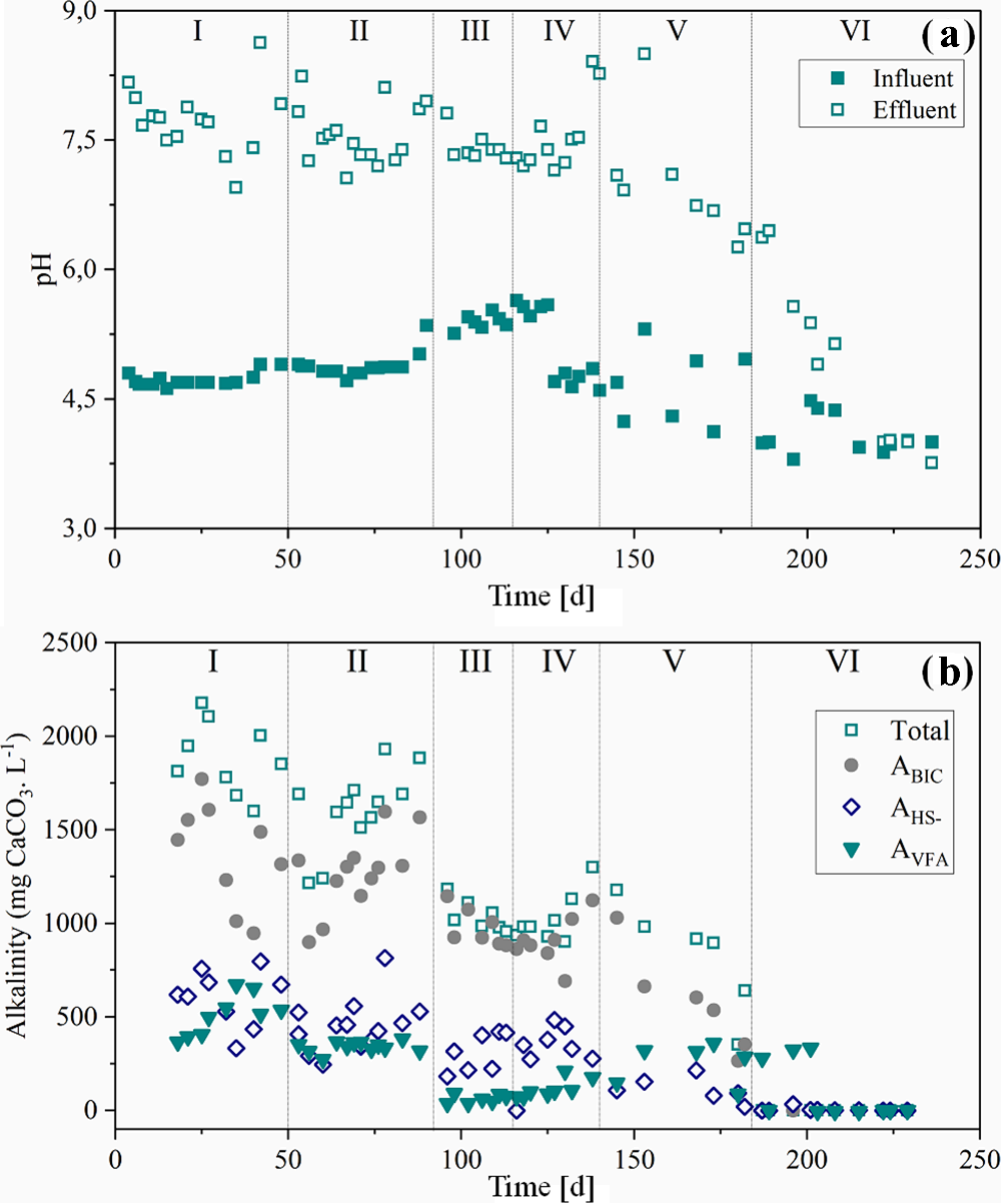
(a) Influent (■) and effluent pH (□); (b) total alkalinity (□), alkalinity due to bicarbonate (●), alkalinity due to HS^–^ (**◊**), and alkalinity due to VFA (▼) during each operational phase

The overall alkalinity and VFA concentrations declined as sulfate and organic matter inputs decreased, mainly due to lower organic and sulfate loading rates. Sulfide contributed 30% to the total alkalinity during Phases I–V. In Phase VI, however, alkalinity due to sulfide dropped to 13%. This reduction was linked to the precipitation of metals as metal sulfides. These reactions lowered sulfide alkalinity and weakened the buffering capacity of the medium, causing a decline in pH.^33^

The intermediate alkalinity/partial alkalinity (IA/PA) ratio averaged 0.6 ± 0.2 in Phase I and 0.5 ± 0.1 in Phase II, stabilizing at 0.3 ± 0.1 in Phases III and IV. In Phase V, the ratio increased to 0.8 ± 0.3, and by day 187 it reached 1.2, indicating acidification of the medium.^77^ This occurred because GOH is rapidly oxidized, leading to its fermentation to the volatile acids production.^78^

Sulfate reduction and COD removal enabled alkalinity generation, increasing the inflow pH from 4.7 to 7.6 in Phase I and from 4.9 to 7.5 in Phase II. In Phases III–V, pH rose from 5.4 to 7.4, from 4.9 to 7.4, and from 3.4 to 6.7, respectively. Regarding effluent pH, no significant differences were observed between Phases I and V (*p*-value > 0.05). After metal addition in Phase VI, the influent pH averaged 4.0 and effluent pH decreased to 4.3. As alkalinity generation declined, the system’s buffering capacity weakened, and during the final 15 days, the effluent pH fell below 4.0, indicating a loss of process stability.

### 3.4 Volatile Fatty Acids Production

Following the complete transition from SV to GOH in Phase V, an increase in the level of VFA production and a decrease in the level of bicarbonate alkalinity were observed. The concentration of VFA increased from 186 mg L^–1^ in Phase III to 233 mg L^–1^ in Phase IV. This value nearly tripled in Phase V, resembling the levels observed in Phases I and II, when the organic load applied was 4.8 ± 0.7 and 3.5 ± 0.2 g L^–1^ day^–1^, respectively.

Chromatographic analyses of the effluent revealed that acetate concentrations predominated in Phases II–VI (Figure 4). Propionate and butyrate were not detected. The dominance of acetate suggests a community enriched in incomplete oxidizers among the SRB, which typically produces acetate as the final metabolite. Similarly, Li et al.^79^ reported that acetate was the most abundant VFA, accounting for 61.0–77.5% of the total, followed by butyrate (18.4–34.0%), propionate (4.1–8.5%), and valerate (0–2.1%) when a COD/SO_4_^2^–^^ ratio of 2.5 was applied.

**Figure 4:**
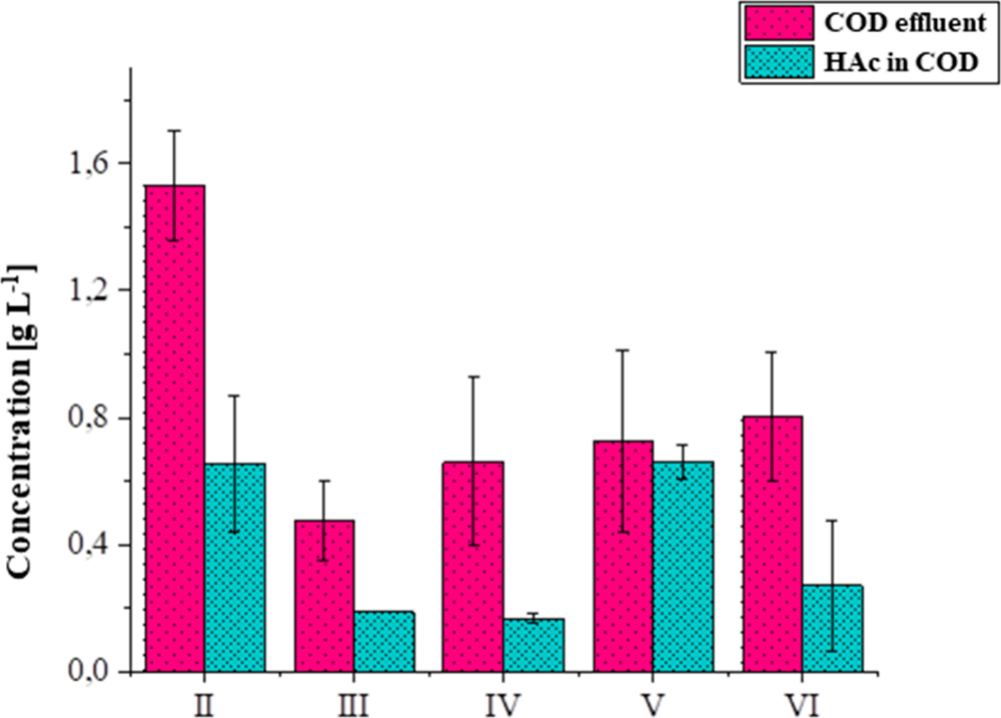
Main concentration (g L^–1^) of COD and volatile fatty acids (HAc in COD basis) detected at the DFSBR effluent during each operational phase.

The absence of propionate may be attributed to the low COD/SO_4_^2^–^^ ratios maintained during these operational phases. Wang et al.^38^ observed that lower COD/SO_4_^2^–^^ ratios promote acetate accumulation while suppressing propionate formation. Propionate serves as both an electron donor and a carbon source for various several SRB genera, including incomplete oxidizers such as *Desulfobulbus* and *Desulfofaba* and complete oxidizers like *Desulfococcus*, *Desulfonema*, and *Desulfosarcina*.^48,80^

Its degradation via the methylmalonyl-CoA pathway, as described for *Desulfobulbus propionicus*, is energetically more favorable than the alternative acrylyl-CoA pathway.^81^ Because the acrylyl-CoA/propionyl-CoA redox couple (+69 mV) requires more energy than the fumarate/succinate couple (+33 mV),^82^ propionate formation and use may be thermodynamically limited under donor-deficient (low COD/SO_4_^2^–^^) conditions. This constraint explains its absence from the reactor effluent.

From Figure 4, it is evident that a portion of the SV, measured as the COD, was not metabolized and thus not converted into organic acids. This unmetabolized fraction may be attributed to the presence of recalcitrant compounds such as phenols and polyphenols.^45^

Based on the average acetate concentration of Phase V effluent, it was possible to estimate the corresponding amounts of oxidized GOH and reduced sulfate via the incomplete pathway. According to Reaction [Disp-formula eqR4] ^83^ and assuming it is the sole metabolic pathway occurring, 1012 mg L^–1^ GOH were consumed, reducing 792 mg L^–1^ sulfate, and resulting in 660 mg L^–1^ acetate. Therefore, 65% of the sulfate reduction occurred via the incomplete pathway and sulfidogenesis accounted for 85% of the oxidized GOH.

R4 $$4C_{3}H_{8}O_{3}+3{{\mathrm{SO}}_{4}}^{2-}+6H^{+}\to 4C_{2}H_{4}O_{2}+4{\mathrm{CO}}_{2}+3H_{2}S+8H_{2}O$$

In Phase VI, the average acetate concentration was 270 mg L^–1^, resulting from incomplete oxidation. Approximately 530 mg L^–1^ sulfate was reduced, of which 200 mg L^–1^ followed the complete oxidation pathway (Reaction [Disp-formula eqR5]), oxidizing 110 mg L^–1^ GOH. This corresponds to 38% of the sulfate reduced via the complete pathway and 62% via the incomplete pathway. Overall, sulfidogenesis accounted for ≈58% of the GOH oxidation in Phase VI.

R5 $$4C_{3}H_{8}O_{3}+7{{\mathrm{SO}}_{4}}^{2-}+14H^{+}\to 12{\mathrm{CO}}_{2}+7H_{2}S+16H_{2}O$$

Under sulfidogenic conditions, SRB does not always utilize GOH directly. Zhou et al.^84^ showed that GOH is first fermented to intermediates such as 1,3-propanediol, ethanol, propionate, H_2_, and formate. SRB then uses these intermediates, particularly 1,3-propanediol and ethanol, as electron donors for sulfate reduction. This highlights the importance of glycerol fermentation in providing substrates that sustain SRB activity.^84^

The decline in the influent pH from Phase IV underscored its importance in stabilizing and enhancing the efficiency of the biological processes involved. By Phase V, inhibition of key microorganisms responsible for converting acids to methane and CO_2_ may have occurred, particularly affecting acetate-consuming methanogens. Consequently, acetate accumulated above 660 mg L^–1^ in Phase V, decreasing the organic matter removal efficiency and internal reactor pH. This acidification, intensified by reduced alkalinity in Phase VI due to metal sulfide precipitation, further lowered the process stability.

Replacing SV, a complex matrix, with GOH, a simpler three-carbon compound, facilitated conversion into shorter-chain acids, altering the metabolic pathways. Acetate, a product of glycerol’s incomplete oxidation by SRB (Reaction [Disp-formula eqR4]), has a p*K*_a_ of 4.75.^85^ Comparing the average acetate concentrations in Phases V (660 mg L^–1^) and VI (270 mg L^–1^) with their respective pH values reveals a marked difference in undissociated acid fractions. At pH 6.7 (Phase V), only 7.6 mg L^–1^ acetate remained undissociated (CH_3_COOH). At pH 5.3 (Phase VI), this value increased to 77.9 mg L^–1^. While low concentrations (≈7 mg L^–1^) are generally tolerated, higher levels (≈78 mg L^–1^) can inhibit microbial metabolism, impairing organic matter degradation, sulfate reduction, and reactor stability.^48,86^

This shift in the balance between dissociated and undissociated acetate can affect cell membrane integrity and metabolic efficiency, especially under moderately acidic conditions. At low pH, nondissociated carboxylic acids diffuse across cell membranes, disrupting internal pH homeostasis and causing cytoplasmic acidification.^46,86^ Reis et al.^87^ reported that 50% of SRB populations were inhibited between pH 5.8 and 7 when exposed to 0.09 mmol L^–1^ (≈54 mg L^–1^) acetic acid. In AMD treatment systems, acetate oxidation to CO_2_ is often the rate-limiting step, requiring extended hydraulic retention times.^46^

This shift in the balance between dissociated and nondissociated acetate forms could influence microbial activity and metabolic efficiency, particularly under moderately acidic conditions. At low pH, nondissociated carboxylic acids diffuse across cell membranes, disrupting internal pH homeostasis and causing cytoplasmic acidification.^46,87^ Reis et al.^88^ observed that 50% of SRB populations were inhibited within a pH range of 5.8–7 when exposed to 0.09 mmol L^–1^ acetic acid (equivalent to 54 mg L^–1^). In AMD treatment systems, the oxidation of acetate to CO_2_ is often the rate-limiting step, requiring extended HRT.^46^

### 3.5 Volatile and Fixed Solids

Figure S1 presents the analysis of fixed solids (FSs) and volatile solids (VSs) from the biomass precipitated and discharged from the reactor bottom. The higher proportion of FS observed in Phases I and II can be attributed to the elevated concentration of SV, which contained a high volatile organic content (VOC).^22^ SV, known for its rich organic load and presence of metals and other inorganic compounds, contributed to this composition.^22^ As the SV input decreased in Phases III and IV, the proportion of FS decreased accordingly. By the start of Phase VI, FS represented 22% of the total solids, increasing to 55% by the end of the operation, indicating the precipitation of metals along with sludge in the reactor.

### 3.6 Microbial Community Analysis

Table 3 summarizes the diversity and richness indices of microbial biomass based on the sequencing data obtained at the end of the DFSBR operation. Despite the challenging conditions (the presence of metals and pH 4.0), the richness indices (Chao1 = 1161), diversity (H′ = 3.519 and Margalef = 77.22), and dominance (Simpson = 0.9088 and *D* = 0.0912) were adequate and comparable to values reported in studies related to AMD or its treatment.^89–91^

**Table 3:** Diversity, Dominance, and Richness Indices for the Bacteria and Archaea Domains of Microbial Biomass at the End of Phase VI of the DFSBR

	general	bacteria domain	archaea domain
Shannon diversity index (H’)	3.519	3.509	1.936
Simpson index (1-D)	0.9088	0.9084	0.75
dominance (D)	0.0912	0.0915	0.25
Margalef index	77.22	74.89	3.206
Chao1 richness index	1161	1117	27.5

A total of 101214 sequencing reads were classified into 891 OTUs. The microbial community was dominated by bacteria, representing 99.8% of the total relative abundance (RA), while archaea accounted for only 0.1%. Within the bacterial domain, 21 phyla were identified, with Firmicutes (63.6%), Bacteroidetes (19.1%), and Proteobacteria (8.9%) being the most prevalent (Figure S2). These phyla are commonly reported as dominant in anaerobic reactors, regardless of reactor configuration or substrate type.^92^ Thermotogae (4.5%) and Spirochaetes (1.3%) also contributed, each exceeding 1% of the total RA.

In the archaeal domain, Euryarchaeota comprised 0.1% of the total RA, while Crenarchaeota represented a minor fraction (0.016%). Despite their low abundance, these archaeal groups are typically involved in key processes such as methanogenesis in anaerobic environments.^93^

At the family level, Veillonellaceae was the most abundant, representing 41.4% of the total relative abundance, followed by Clostridiaceae (13.1%), Bacteroidaceae (13.0%), and Desulfovibrionaceae (5.3%). In total, 154 families were classified, with those representing less than 1.0% of the total abundance combined accounting for 8.5%.

The predominant genera identified were *Bacteroides*, *Clostridium*, *Pectinatus*, *Zymophilus*, *Desulfovibrio*, *Geobacter*, and *Acidaminococcus*, which together accounted for 79.6% of the total community. *Zymophilus* (RA of 24.9%), from the Veillonellaceae family, is strictly anaerobic and fermentative, commonly found in sulfidogenic environments and enriched sediments (Figure 5).^86,94^

**Figure 5:**
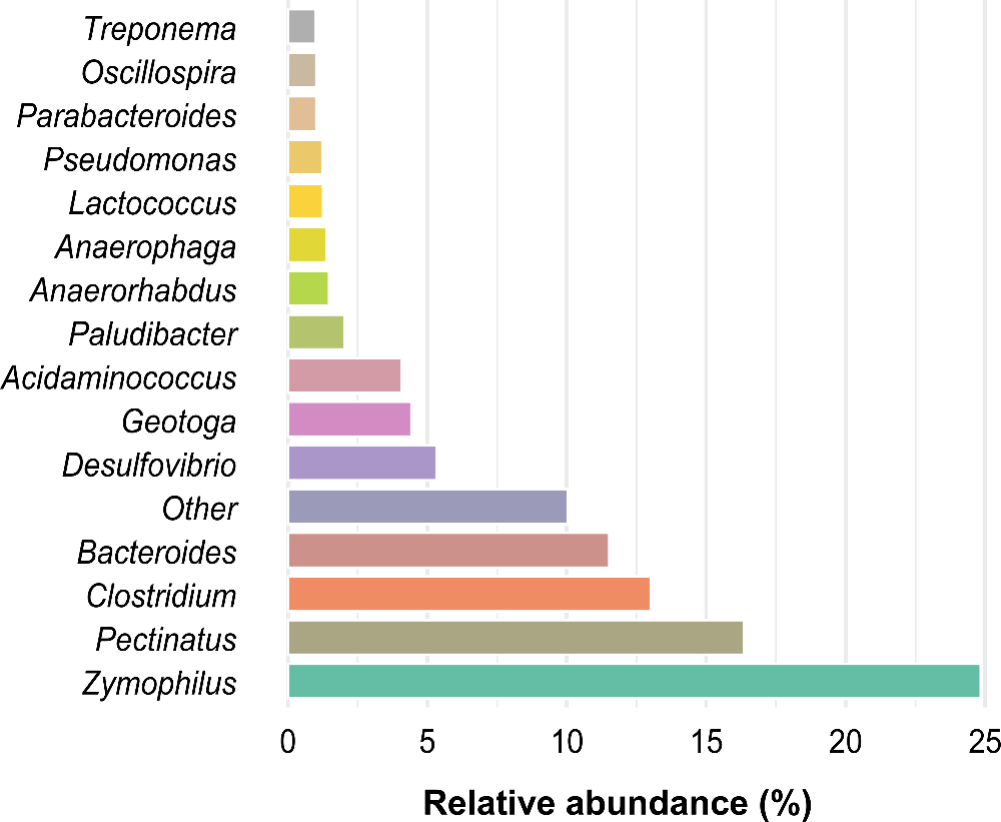
Relative abundance of the main genera identified in the biomass retrieved from the support material at the end of the DFSBR operation.

The genus *Geotoga* (4.4% RA) includes anaerobic, fermentative, and sulfur-reducing bacteria that produce H_2_, CO_2_, acetate, and ethanol.^95^ *Geotoga*’s abundance in sulfidogenic bioreactors can vary significantly depending on the carbon source: e.g., 17.2% with glucose and only 0.2% with formate,^96^ highlighting the influence of carbon sources on microbial composition.

*Pectinatus* (16.4% RA) are fermentative, H_2_S-producing bacteria that convert glucose and fructose into acetate and propionate.^97^ Although often associated with beer spoilage, there are no previous reports of *Pectinatus* in AMD or sulfidogenic reactors.

SRB accounted for approximately 7.0% of the community and included genera such as *Desulfovibrio* (5.3%), *Desulfobulbus*, *Desulfatibacillum*, *Desulfomicrobium*, *Desulfosporosinus*, and *Desulfobacter*. The metabolism of sulfate-reducing microorganisms can be categorized into assimilatory sulfate reduction (ASR) and dissimilatory sulfate reduction (DSR) pathways.^38,98^ The ASR pathway is primarily responsible for incorporating sulfide as an intermediate in the biosynthesis of sulfur-containing amino acids, such as cysteine and methionine.^38,79^ In contrast, the DSR pathway operates under anaerobic conditions, where sulfate serves as a terminal electron acceptor and is reduced to sulfide as the final product.^98^

*Desulfovibrio*, a mesophilic δ-proteobacterium and an incomplete organic oxidizer,^48,99^ exhibits higher sulfate affinity and growth rates than other SRB, such as *Desulfobulbus* and *Desulfobacter*.^100^ Its RA is sensitive to pH changes; for example, Hidalgo-Ulloa et al.^101^ observed a drop from 5.1 ± 0.8% at pH 6.9 to undetectable levels at pH 3.8. In contrast, Menezes et al.^17^ showed its dominance in a thermophilic (55 °C) anaerobic fluidized bed reactor co-digesting SV and GOH. Santos et al.^102^ and Wang et al.^38^ identified *Desulfovibrio* as the main SRB genera contributing to sulfate reduction processes in the analyzed system. These microorganisms possess key functional genes, such as DsrAB, which are widely used as molecular markers to assess the diversity and abundance of DSR-capable populations in different environments.^98^ Notably, the expression of DsrAB genes is highest when the COD/SO_4_^2^–^^ ratio is around 1.0, indicating an optimal condition for sulfate-reducing activity.^98^

Recent genomic and metatranscriptomic studies have provided detailed insights into of the DSR pathway, providing a molecular framework to interpret the functional potential of sulfate-reducing communities in anoxic systems.^103,104^ The canonical DSR pathway involves a cascade of enzymatic reactions encoded by sulfate adenylyltransferase (sat), adenylyl sulfate reductase alpha and beta subunits (aprAB), and dissimilatory sulfite reductase alpha and beta subunits (dsrAB), which convert sulfate to sulfide. The dsrAB genes encode the catalytic subunits of the dissimilatory sulfite reductase, while the dsrMKJOP (transmembrane complex involved in electron transport) complex functions as the terminal reductase that mediates electron transfer from the cytoplasmic membrane to the dsrAB enzyme complex.^103^ The identification of genera such as *Desulfovibrio* in the present study suggests that this canonical sat–aprAB–dsrAB–dsrMKJOP pathway was likely active during the co-digestion of SV and GOH, consistent with the high sulfate removal efficiency (99%) and the concurrent generation of H_2_S and alkalinity.

The presence of fermentative taxa including *Clostridium*, *Bacteroides*, and *Zymophilus* indicates that the microbial consortium established a syntrophic network, where GOH and complex organics from SV were fermented into intermediates such as acetate and propionate.^105–108^ These compounds can serve as electron donors for SRB through dsrAB-mediated sulfate reduction, linking carbon fermentation to sulfur respiration. This coupling is consistent with the observed acetate accumulation and bicarbonate production, which together contributed to pH buffering within the reactor.

Although functional gene quantification was not performed, it is plausible that the dominant Desulfovibrionaceae and Desulfobacteraceae members carried the dsrAB operon along with regulatory genes such as dsrC, dsrD, and dsrT, which participate in substrate delivery and redox regulation.^104^ The downregulation of dsrD observed under high sulfide concentrations^109^ could explain the moderate sulfide accumulation detected at the end of the operational phases, suggesting a feedback mechanism that limits excessive H_2_S formation. In parallel, the low abundance of methanogens inferred from 16S rRNA sequencing implies reduced expression of mcrA, the key gene encoding methyl coenzyme-M reductase, which catalyzes the final step of methanogenesis.^92^ This aligns with the acidogenic and sulfidogenic dominance observed, where sulfate reducers outcompete methanogens for available electron donors under low-pH and metal-rich conditions.

Fermentation products such as acetate and propionate are preferentially consumed by SRB, which generally prefer propionate over butyrate.^110^ In contrast, acetoclastic methanogens prefer acetate. Thus, the observed accumulation of undissociated acetate in Phase VI may be linked to the low abundance of archaea.

The microbial community identified in this study underscores the adaptability of anaerobic systems under challenging conditions, including low pH and the presence of metals. The performance and stability of pollutant removal systems can be compromised by elevated concentrations, toxicity, or complex contaminant mixtures. However, such limitations can be addressed by optimizing the operational parameters and enhancing the microbial resilience. Strategies such as microbial acclimatization or bioaugmentation can improve system robustness and pollutant removal efficiency.^111^ Additionally, the shifts in microbial diversity and relative abundance observed in this study, compared to previous reports, further highlight the influence of key operational factors, particularly pH and substrate composition, in shaping the microbial community structure and driving functional performance.

## 4 Conclusions

This study successfully demonstrated the technical viability and sustainability of anaerobically co-digesting SV and GOH as cost-effective electron donors for the treatment of sulfate-rich and acidic wastewaters such as AMD. The combination of these substrates within a single sulfidogenic bioreactor achieved up to 99% sulfate reduction and substantial alkalinity generation, mitigating the need for external alkalinizers. This crucial mechanism neutralized the effluent’s acidity (pH 4.9–7.4) and facilitated the efficient precipitation of metals. The syntrophic interactions established between fermentative and SRB—mainly *Zymophilus*, *Clostridium*, and *Desulfovibrio*—proved essential to sustaining sulfidogenesis under acidic and metal-rich conditions, highlighting the microbial resilience and functional redundancy of these communities even at pH < 5.0. Beyond empirical performance, this work demonstrated a systemic innovation: transforming two waste streams from the biofuel industry into functional reagents that enhance the sustainability and circularity of mine wastewater treatment. The rational use of SV and GOH promotes regional resource synergy, reducing both chemical and transportation costs.

## Supplementary Material



## Notes

Tables summarizing organic and sulfate loads removed in each operational phase and characterization of sugarcane vinasse; figures showing proportion of fixed and volatile solids determined from sludge gravimetric analysis and relative abundance of the main microbial taxa identified in retrieved biomass (PDF)
