# Supplementary material for: Sugarcane Vinasse and Glycerol as Potential Carbon Sources for Sulfate-Rich Wastewater Treatment: Mitigating the Impacts of Acid Mine Drainage
Source: ACS Omega. 2025 Dec 8;10(50):61679–92. doi: 10.1021/acsomega.5c07834 (PMC12750253; doi:10.1021/acsomega.5c07834)
Supplement: Supplementary file 1 [file ao5c07834_si_001.pdf]

Supplementary Information

Sugarcane Vinasse and Glycerol as potential carbon sources for Sulfate-Rich Wastewater Treatment: Mitigating the Impacts of Acid Mine Drainage

Elis W. Nogueira[a], Leandro A. G. Godoi[a], Pamela T. Couto[a], Rodrigo B. Carneiro[a,c*], Paula Y. Takeda[a], Mirabelle P. Cunha[a], Bruna D. B. Zampieri[b], Gunther Brucha[b], Marcia H. R. Z. Damianovic[a]

[a] Biological Processes Laboratory, São Carlos School of Engineering, University of São Paulo, Avenida João Dagnone 1100, São Carlos, 13563-120, SP, Brazil, email: watanabeelis90@gmail.com; leandro.godoi86@gmail.com; pamela.couto@usp.br; pytakeda@usp.br; mirabelle.cunha@gmail.com; mdamianovic@sc.usp.br

[b] Anaerobic Biotechnology Laboratory, Science and Technology Institute, Federal University of Alfenas (UNIFAL-MG), Rodovia José Aurélio Vilela, 11999 (BR 267, Km 533), Poços de Caldas, 37715-400, MG, Brazil, e-mail: brunadbzampieri@gmail.com; gunther.brucha@gmail.com

[c] Laboratory of Chromatography, São Carlos Institute of Chemistry (IQSC), University of São Paulo (USP), Avenida Trabalhador São-Carlense 400, São Carlos, 13566-590, SP, Brazil, email: rodrigocarneiro@sc.usp.br

*Corresponding author Tel: +55-16-99774-4044 ORCID: 0000-0002-9188-4133 rodrigocarneiro@sc.usp.br / rodrigobrazcarneiro@yahoo.com.br

Table S1 Organic and sulfate loads removed in each phase.

**Phases**	**I**	**II**	**III**	**IV**	**V**	**VI**
COD load removed (g COD L^{-1} d^{-1})	2.8 ± 0.4	1.8 ± 0.3	1.0 ± 0.5	1.1 ± 0.3	1.6 ± 0.3	0.7 ± 0.4
COD removal (%)	58 ± 10	51 ± 6.0	63 ± 10	61 ± 15	67 ± 12	35 ± 18
Sulfate load removed (g SO_4 L^{-1} d^{-1})	1.6 ± 0.3	1.5 ± 0.2	1.2 ± 0.5	1.4 ± 0.6	1.4 ± 0.4	0.6 ± 0.5
Sulfate removal (%)	80 ± 8.0	75 ± 6.0	90 ± 4.0	99 ± 1.0	82 ± 11	34 ± 23

Table S2 Composition of raw SV used in Phases I, II, III, and IV

**Constituents**	**Concentrations**
**Organic matter**	
COD (mg O_2 L^{-1})	120,000
BOD (mg O_2 L^{-1})	60,600
Soluble carbohydrates (mg L^{-1})	17,200
Total volatile acids (mg CH_3COOH L^{-1})	9,500
Total phenols (mg L^{-1})	12
**Nutrients**	
Sulfate (mg SO_4^{2-} L^{-1})	25,200
Total nitrogen (mg TKN L^{-1})	3,100
Ammoniacal nitrogen (mg NH_4 L^{-1})	400
Phosphorus (mg PO_4^{3-} L^{-1})	860
**Metals**	
Potassium (mg L^{-1})	4,000
Calcium (mg L^{-1})	2,200
Sodium (mg L^{-1})	6,000
Magnesium (mg L^{-1})	920
Manganese (mg L^{-1})	10.2
Zinc (mg L^{-1})	1.2
Copper (mg L^{-1})	1.1
Lead (mg L^{-1})	1.0
Nickel (mg L^{-1})	0.6
Cadmium (mg L^{-1})	0.2
Iron (mg L^{-1})	40
**Solids**	
Total solids (g L^{-1})	103
Volatile total solids (g L^{-1})	71
Total suspended solids (g L^{-1})	20
Volatile suspended solids (g L^{-1})	18
Total dissolved solids (g L^{-1})	83
Volatile dissolved solids (g L^{-1})	53

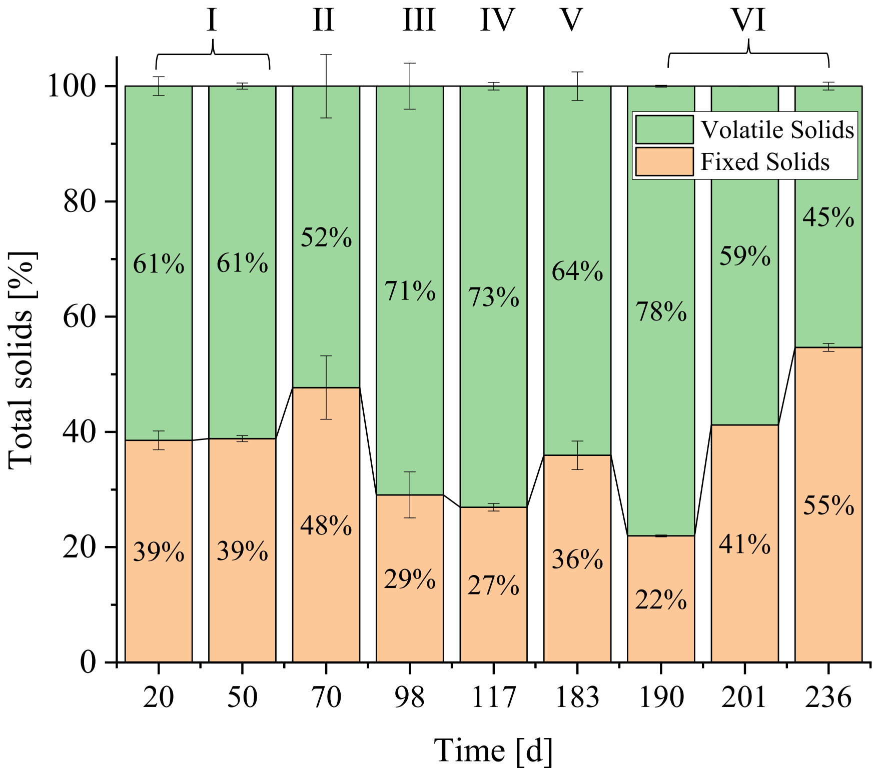

Figure S1 Proportion of fixed and volatile solids corresponding to the gravimetric analysis of the sludge.

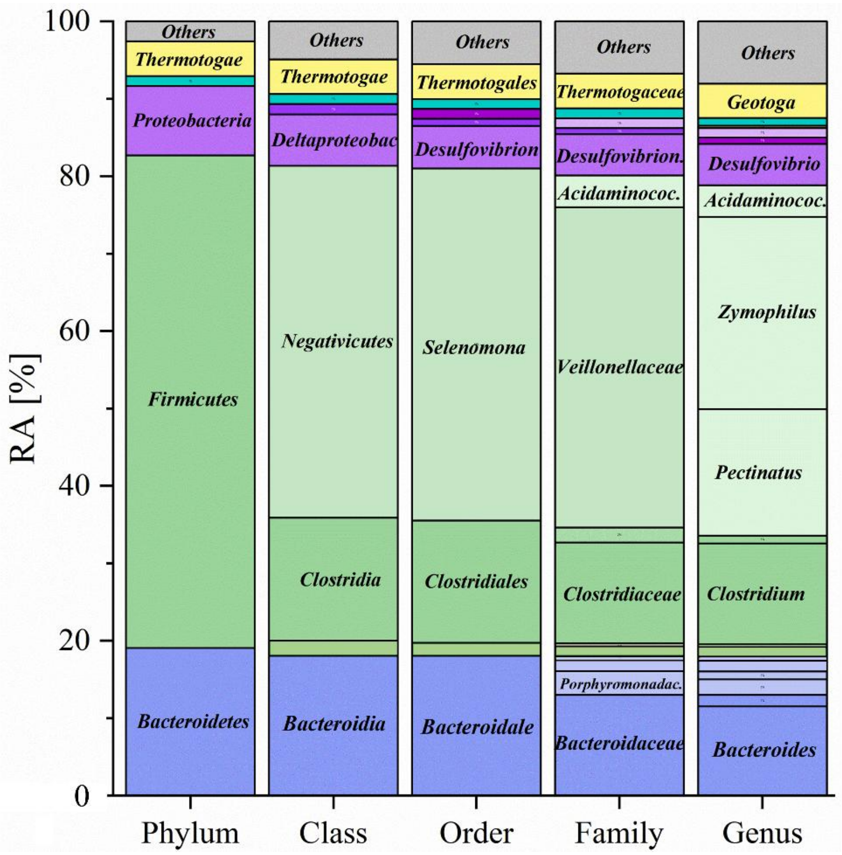

Figure S2 Relative abundance of the main phyla, classes, order, families and genera identified in the biomass retrieved from the support material at the end of DFSBR operation.
